# Exosomal ERp44 derived from ER-stressed cells strengthens cisplatin resistance of nasopharyngeal carcinoma

**DOI:** 10.1186/s12885-021-08712-9

**Published:** 2021-09-08

**Authors:** Tian Xia, Hui Tian, Kaiwen Zhang, Siyu Zhang, Wenhui Chen, Si Shi, Yiwen You

**Affiliations:** 1 Department of Otorhinolaryngology Head and Neck surgery, Affiliated Hospital of Nantong University, Nantong, Jiangsu Province China; 2 Institute of Otorhinolaryngology Head and Neck Surgery, Affiliated Hospital of Nantong University, Nantong, Jiangsu Province China; 3 Medical College of Nantong University, Nantong, Jiangsu Province China

**Keywords:** Nasopharyngeal carcinoma, ERp44, ER stress, Exosomes, Chemoresistance

## Abstract

**Background:**

Nasopharyngeal carcinoma (NPC) is one of the most common malignancies in head and neck. Platinum-based chemotherapy is an important treatment for NPC. However, the molecular mechanism of resistance to platinum drug remains unknown. Endoplasmic reticulum resident protein 44(ERp44), an unfolded protein response (UPR)-induced endoplasmic reticulum(ER) protein, is induced during ER stress. This research explored the mechanism of ERp44 in strengthening cisplatin resistance in NPC.

**Methods:**

Western blot and immunohistochemistry were used to investigate the expression of ERp44 and Glucose-Regulated Protein 78(GRP78) in NPC. We took CCK8 to detect the role of ERp44 on cell chemosensitivity. Flow cytometric analysis and western blot were taken to analyze cell apoptosis. We performed differential centrifugation to isolate exosomes from serum or conditioned media of cells and analyzed the impact of exosomal ERp44 on cells cisplatin sensitivity. Finally, the results were confirmed in vivo.

**Results:**

We found the increased expression of ERp44 and GRP78 in NPC and ERp44 was highly expressed in ER-stressed tissues. Cell proliferation was inhibited after cisplatin treatment when ERp44 was knocked down and ERp44 strengthened cisplatin resistance by influencing cell apoptosis and pyroptosis. Then we also collected exosomes and cell viability was increased after the addition of NPC-derived-exosomes with cisplatin treatment. More importantly, our results showed under ERS, NPC cells secreted exosomes containing ERp44 and could transfer them to adjacent cells to strengthen chemoresistance.

**Conclusion:**

Our data suggested that exosomal ERp44 derived from ER-stressed NPC cells took an inevitable role in NPC chemoresistance and might act as a treatment target.

## Background

Nasopharyngeal carcinoma (NPC), characterized by its unique distribution, is prevalent in east and southeast Asia [1]. There are over 130,000 NPC new cases reported in 2020 [2]. More than 70% of the patients are classified as locoregionally advanced while being diagnosed [3]. Now concurrent chemoradiotherapy has been the standard treatment for locoregionally advanced NPC and could improve patients’ survival [4]. However, resistance to chemotherapy is still a major problem for treatment failure [5]. NPC patients are sensitive to chemotherapy in the initial but then they might acquire resistance, which will cause the failure of treatment [6]. As cisplatin is commonly a first choice for chemotherapy, determining the mechanism contributing to cisplatin resistance will help us improve treatment efficiency.

Endoplasmic reticulum (ER), commonly known as a significant component of endomembrane system, is responsible for the regulation of lipid, glucose, Ca^2+^ homeostasis and protein synthesis [7]. In some conditions, especially in tumor microenvironment, cells undergo nutrient deprivation, hypoxia or drug-induced toxicity, protein folding is interfered. More and more misfolded proteins accumulate in the lumen of ER to cause a state of “ER stress”. ER stress could activate Unfolded Protein Response (UPR) to defense the damage [8, 9]. Studies report that ERS is activated in malignances and contributes to several aggressive characteristics [10, 11]. ERS could also influence chemoresistance. It has been reported that UPR activation is correlated with chemotherapy resistance in osteosarcoma, breast cancer and so on [12, 13]. Glucose-Regulated Protein 78 (GRP78), a major molecular chaperone protein in the ER, was correlated with malignant behaviors of tumors and could act as an important ERS biomarker [14, 15]. GRP78 could increase ionizing radiation and cisplatin resistance in NPC cells [16, 17], but the detailed molecular mechanism still need to be further clarified.

The ER resident protein 44(ERp44), a UPR-induced ER protein of the protein disulfide isomerase (PDI) family, is induced during ERS. It regulates Ca^2+^ signaling, protein folding and homeostasis in the ER [18]. With its indispensable function, it takes important roles in tumor progression. Aberrantly expression of ERp44 was reported in breast cancer, colorectal cancer, oral squamous cancer and might act as a prognostic biomarker [19–21]. In our previous research, we have also reported that ERp44 was highly expressed in NPC and associated with patients’ survive state and clinical stages, it also participated in promoting cells proliferation and migration [22]. However, the role of ERp44 on cell chemoresistance remains unclear.

Exosomes are discovered as a new system for cell-to-cell communication nowadays [23]. They are 40 to 100 nm double-layer membrane extracellular vesicles and could carry proteins, RNAs, DNAs to recipient cells. Exosomes could influence the biological functions of tumors [24, 25]. Our previous studies showed that NPC-derived exosomes played important roles in mediating angiogenesis and might be a tissue-based marker for NPC [26]. Increasing evidence also highlighted the significance of exosomes in drug resistance and they could transfer contents to recipient cells to confer chemoresistance [27]. Under ERS, tumor cells could secrete exosomes to influence tumor progression. In liver cancer, ERS promoted immunosuppression of macrophages by releasing exosomes [28, 29]. Nevertheless, whether exosomes released by ER stressed-NPC cells could influence cells chemosensitivity needs to be further investigated.

In the present research, we investigated the role and molecular mechanism of ERp44 on cell chemosensitivity. We found ERp44 was highly expressed in ER-stressed tissues and could reduce cisplatin sensitivity by influencing cell apoptosis and pyroptosis. More importantly, under ERS, NPC cells produced ERp44-containing exosomes and could transfer them to adjacent cells to strengthen chemoresistance. These results suggested that ERp44 takes inevitable roles in NPC chemoresistance and might act as a treatment target.

## Methods

### Human NPC specimens and immunohistochemistry

Paraffin-embedded NPC specimens and fresh biopsy samples were obtained from Affiliated Hospital of Nantong University. Tumor samples were confirmed by pathological diagnosis as nasopharyngeal squamous carcinoma. The research got approval from Ethics Committee of Affiliated Hospital of Nantong University (Ethical batch number:2018-L049). Immunohistochemistry (IHC) was carried out and evaluated as previously described [30]. Slides were incubated with anti-GRP78(11587–1-AP, Proteintech) and anti-ERp44 (16016–1-AP, Proteintech).

### Cell culture and transfection

NPC cells CNE2(low differentiation) and 5-8F (high tumorigenesis and high metastasis) were generously gifted by Sun Yat-Sen University and Xiang-Ya School of Medicine. Cells were growing in RPMI 1640 (Biological Industries Israel Beit-Haemek, 01–100-1ACS) with10% fetal bovine serum (Biological Industries Israel Beit-Haemek, 04–001-1ACS). We obtained shRNAs from Shanghai Genechem Co, Ltd. shERp44–1, forward sequence 5′- GATCCCGCACCCAGTGAATATAGGTATCTCGAGATACCTATATTCACTGGGTGCTTTTTGGAT-3′, shERp44–2, forward sequence 5′- GATCCCGCTCGGCAATTAATAAGTGAACTC GAGTTCACTTATTAATTGCCGAGCTTTTTGGAT- 3′, shERp44–3, forward sequence 5′- GATCCCCCGATG TCATTAAGGAAGAATCTCGAGATTCTTCCTTAA TGACATCGGTTTTTGGAT-3′. Tumor cells were seeded on plates at an appropriate density and transfected with shRNAs by Lipofectamine 2000 (Invitrogen, USA) according to the instructions.

### CCK8

Cell counting kit-8 (Beyotime Institute of Biotechnology, China) was used to measure cell proliferation. 1 × 10^4^ cells transfected with ERp44-shRNA or control were seeded into a 96-well plate (Corning inc, Corning NY). After cells adhered, 20 μg/ml cisplatin was added and treated cells for different hours. 10 μl CCK-8 was added to each well and incubated for 1.5 h. A microplate reader (F-2500 Fluorescence Spectro-photometer, Hitachi) was used to measure the absorbance at 450 nm.

### Western blot and quantitative RT-PCR

We extracted proteins from tissues, cells and exosomes. Bicinchoninic acid (BCA) protein assay kit (23,227, ThermoFisher Scientific, USA) was used to quantify protein concentration. Western blot was taken to detect protein expression as previously described [31]. Anti-GRP78(11587–1-AP), anti-ERp44 (16016–1-AP), anti-caspase3(19677–1-AP) were obtained from Proteintech. Anti-GSDME (215191, 221843) were obtained from Abcam. Anti-NF-κB (4764), anti-p-NF-κB (3033), anti-Bax (2772), anti-Bcl-2(2872), anti-Bcl-xl (2764) were obtained from Cell Signaling Technology. qRT-PCR was used to detect ERp44 mRNA expression after the transfection as previously described [22]. The primers sequences of ERp44 were as follows: forward: 5′-CCTGTGCCAGGCCTCAATAC − 3′, reverse: 5′-TGGCACTGGGCTTCCTGATA − 3′. We normalized the data with GAPDH.

### Extraction and characterization of exosomes

Exosomes were extracted from serum or cells culture medium by differential ultracentrifugation as previously described [26]. To characterize the exosomes, we fixed them with 2.5% glutaraldehyde and then took ultracentrifugation. After added to a formvar/carbon-coated grid and negatively stained with 3% aqueous phosphotungstic acid, exosomes were observed under Transmission electron microscopy (TEM) (JEM-1230, JEOL, Tokyo, Japan). Nanoparticle tracking analysis (NTA) was taken to further confirm exosomes, we took the NanoSight NS300 (Malvern) for real-time observation. The data was analyzed by NTA software version 3.2.

### Cellular uptake of exosomes

Purified exosomes were resuspended and treated with PKH-67 dye diluted in diluent C. We then took ultracentrifugation to collect PKH-67 labeled exosomes. After incubating with exosomes for 2 h, cells were fixed in 4% paraformaldehyde and nuclei were stained with Hoechst. Cellular uptake was observed with a TCS SP-5 confocal microscope (Leica Microsystems, Wetzlar, Germany).

### Nuclear morphometry

We seeded cells into a 24-well plate overnight and fixed them with 4% paraformaldehyde. Then cells were treated with 0.5% of Triton X-100. After adding Hochest to dye cells nuclear, we observed the nuclear morphology with fluorescence microscope. Apoptotic cells were identified by nuclei pyknosis.

### Cell apoptosis assay

We evaluated cell apoptosis with Annexin V-PE Apoptosis Detection Kit (BD Biosciences, Oxford, UK). Cells were collected and resuspended at a concentration of 1 × 10^6^ cells/ml. Then we transferred 100 μl solution (1 × 10^5^ cells) to a culture tube. After adding 5 μl of Annexin V and 5 μl 7-AAD, cells were incubated for 15 min without light. Then 400 μl 1 × Binding Buffer was added and apoptotic cells were determined by flow cytometry.

### In vivo assay

We used 5-week-old BALB/c nude mice (Laboratory Animal Center of Nantong University, Nantong, China) to assess the role of ERp44 on NPC chemosensitivity in vivo. They were housed in laminar shelves without specific pathogen under proper temperature and humidity and fed with aseptic water and feed. The study was approved by the Animal Ethics Committee of Nantong University (RDD number: 20180227–008).

Briefly, we randomly divided mice into 6 groups (5 in each group). 1 × 10^6^ CNE2 cells transfected with shERp44 or control were subcutaneously injected into the mice. After tumor formation, we intraperitoneal injected cisplatin every 2 days. The last two groups represented that CNE2 cells were subcutaneously injected into nude mice, after tumor formation, shERp44-exosomes or NC-exosomes were intratumorally injected every 2 days. The weight was measured every 2 days and all the mice were sacrificed 3 weeks after inoculation by cervical dislocation after anesthetization. The tumors were removed and fixed in formalin or − 80 °C for further research. After the experiment, nude mice were packed and disposed to a specific fridge for further harmless disposal. The experiments were followed NIH Guidelines and were approved by the Administration Committee of Experimental Animals, Jiangsu Province, China (Approval ID:SYXK(SU)2007–0021).

### GEPIA2 and UALCAN analysis

We took biological information web tools to analyze ERp44 and GRP78 mRNA expression in Head and Neck squamous cell carcinoma (HNSC). The expressions of GRP78 (HSPA5) and ERp44 were evaluated in HNSC tissues from TCGA (T = 520; *N* = 44). GEPIA2(Gene Expression Profiling Interactive Analysis) database (http://gepia2.cancer-pku.cn/) was used to detect mRNA expression. UALCAN(http://ualcan.path.uab.edu/) was used to detect the correlation between ERp44 and GRP78 expression.

### Statistical analysis

We repeated experiments in triplicate and statistical analysis were performed by One-way ANOVA and two-tailed student’s t-tests with SPSS17.0. Results were presented as means±standard deviations and *P*<0.05 was considered statistically significant.

## Results

### ERp44 was highly expressed in ER-stressed tissues

**Fig. 1:**
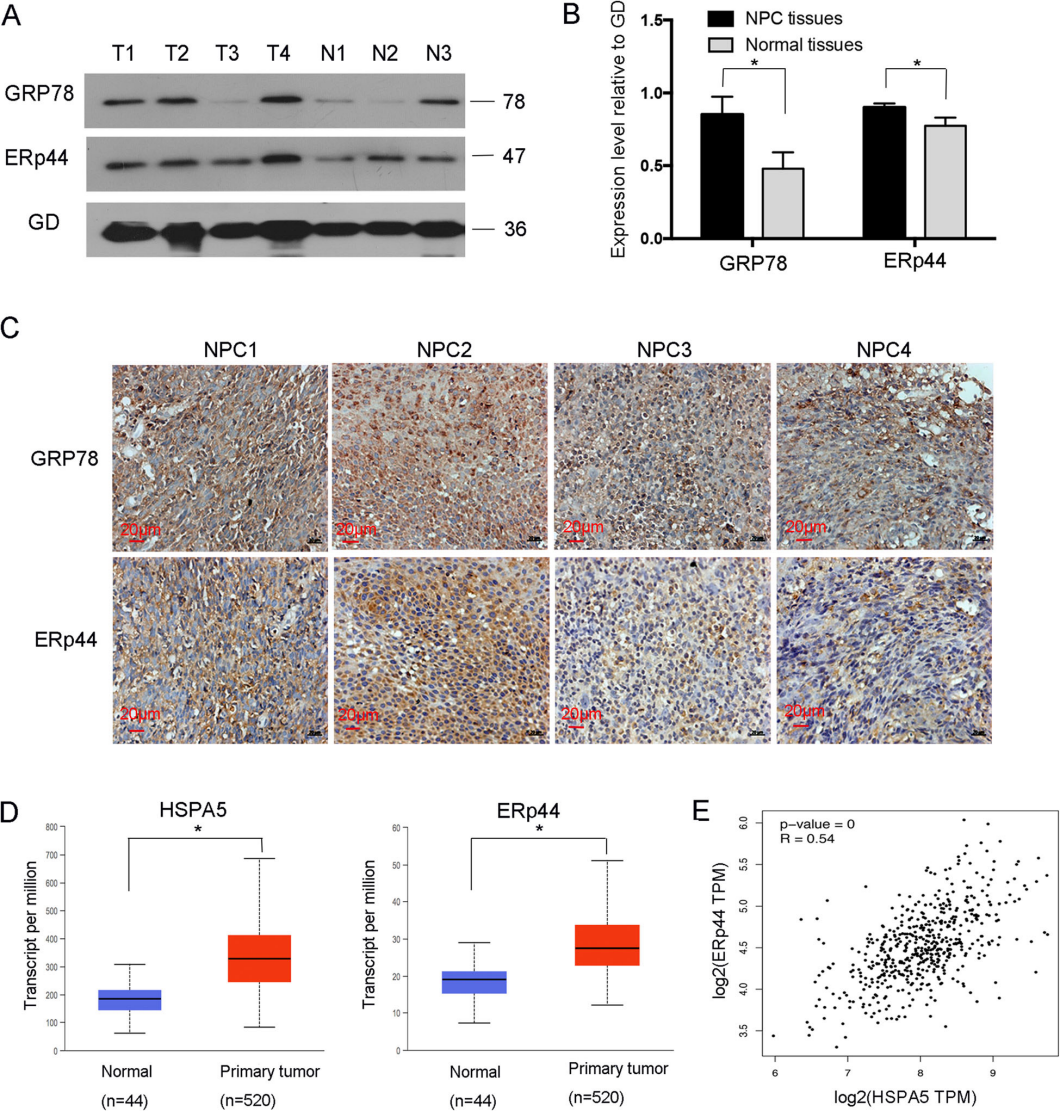
ERp44 was highly expressed in ER-stressed tissues. **A**: Western blot was used to detect GRP78 and ERp44 expressions in NPC and normal tissues. T: Nasopharyngeal squamous cell carcinoma tissues. N: Nasopharyngeal epithelium tissues. **B**: The histogram showed GRP78 or ERp44 expression level relative to GAPDH (GD) by densitometry. **C**: The representative images of immunohistochemical detection of GRP78 or ERp44 in NPC tissues. **D**: The expressions of GRP78 (HSPA5) and ERp44 in HNSC tissues from TCGA (T = 520; *N* = 44). The results were obtained from the GEPIA2 web tool. **E**: The correlation of GRP78(HSPA5) and ERp44 expression in HNSC tissues from TCGA. The results were obtained from the UALCAN web tool. *P < 0.05

First of all, we measured the expression of ERS-related marker GRP78 in NPC. Western blot showed that among four NPC tissues, three of them had highly expression of GRP78 (Fig. 1A). ERp44 took important roles in ERS, and it was overexpressed in NPC than normal tissues (Fig. 1A). More importantly, in tissues which had highly expression of ERp44 also had elevated GRP78 (Fig. 1A-C). From Fig. 1C, IHC data showed that ERp44 and GRP78 were predominantly detected in cytoplasm and an obvious increase of ERp44 was noted in ER-stressed tissues. To further confirm our results, we analyzed high throughput HNSC RNA expression profile datasets from The Cancer Genome Atlas (TCGA), and found GRP78 and ERp44 were highly expressed in HNSC tissues (Fig. 1D). What’s more, GRP78 was correlated with the expression of ERp44 (Fig. 1E). Taken together, ERp44 was up-regulated in NPC and positively correlated with the expression of GRP78.

### ERp44 took an important role in chemosensitivity of NPC cells

**Fig. 2:**
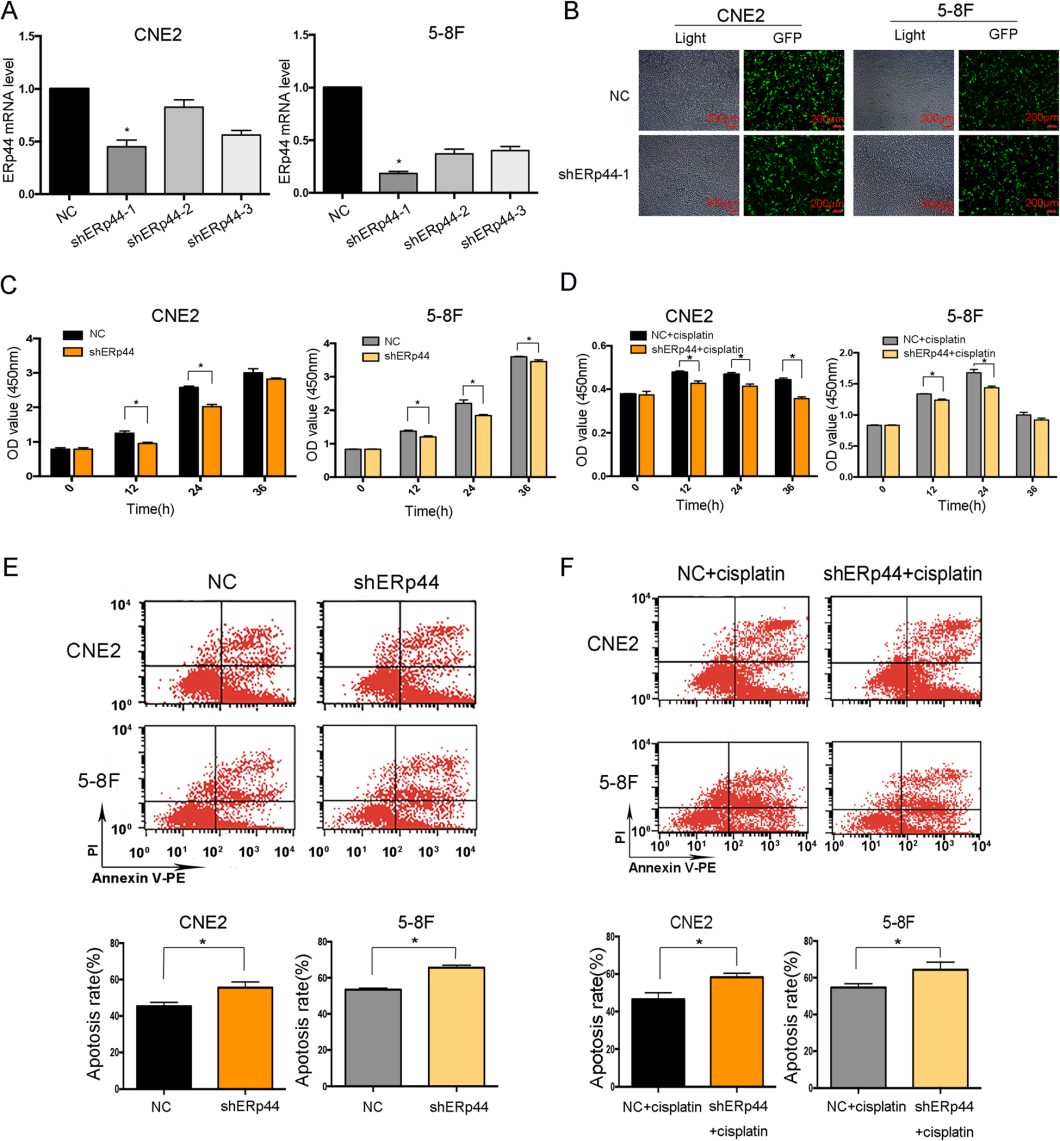
ERp44 took an important role in chemosensitivity of NPC cells. **A**: NPC cells (CNE2 and 5-8F) were transfected with shERp44 (1, 2, 3). qRT-PCR was used to measure mRNA level of ERp44. **B**: The transfection efficiency of NPC cells was captured by fluorescent microscope. **C**: CCK8 assay was taken to investigate cell proliferation after ERp44 was knocked down. **D**: CCK8 assay was taken to investigate cell proliferation with the addition of cisplatin. **E**: Flow cytometry apoptosis experiment was taken to investigate cell apoptosis after ERp44 was knocked down. **F**: Flow cytometry apoptosis experiment was taken to investigate cell apoptosis with the addition of cisplatin. *P < 0.05

Studies have reported that ERS leads to drug resistance. As ERp44 was highly expressed in ER-stressed tissues, we hypothesized it might also influence cells chemosensitivity. We chose CNE2 and 5-8F NPC cells for further studies as CNE2 was the most common pathological pattern of NPC and 5-8F had characteristics of high tumorigenesis and high metastasis. We transfected NPC cells (CNE2,5-8F) with shRNAs and found shERp44–1 was the most effective one (Fig. 2A-B). CCK8 assay showed when ERp44 was down-regulated, cell proliferation was inhibited (Fig. 2C). Next, the influence of ERp44 on drug resistance was examined. We found cell viability was decreased when ERp44 was knocked down with cisplatin treatment (Fig. 2D). When cells undergo drug resistance, they show the characteristics of preventing apoptosis. Our results showed apoptosis cells were increased in ERp44 low-expression groups after cisplatin treatments (Fig. 2E-F). So ERp44 took an important role in chemosensitivity of NPC cells.

### ERp44 reduced cisplatin sensitivity by influencing cell apoptosis and pyroptosis

**Fig. 3:**
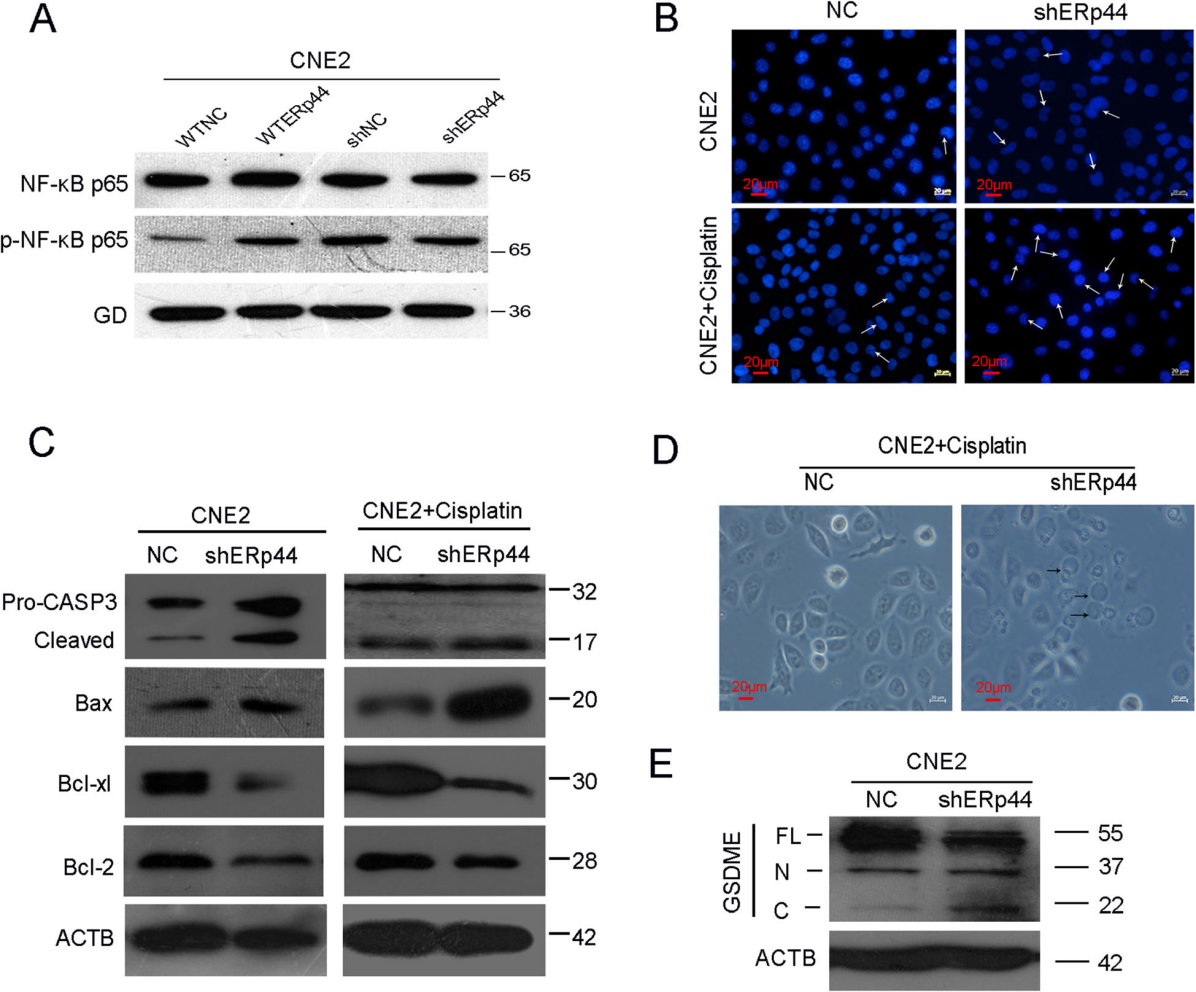
ERp44 reduced cisplatin sensitivity by influencing cell apoptosis and pyroptosis. **A**: Western blot was used to detect p-NF-κB expression after ERp44 was knocked down. **B**: Morphological characteristics of nuclear pyknosis was observed under fluorescent microscope. **C**: Western blot was used to detect the expression of apoptosis markers after ERp44 was knocked down with cisplatin treatment. **D**: Morphological characteristics of pyroptosis was observed under microscope after ERp44 was knocked down with cisplatin treatment. **E**: Western blot was used to detect GSDME expression after ERp44 was knocked down

Transcription factor nuclear factor kappa-B (NF-κB) is involved in cell apoptosis and platinum-based chemotherapy resistance [32]. We confirmed that in CNE2, when ERp44 was overexpressed, p-NF-κB was increased, while when ERp44 was downregulated, it was decreased (Fig. 3A). Next, we took research to observe cell apoptosis during chemotherapy. There were more cells present nuclear pyknosis after transfected with shERp44, and apoptosis cells were further increased with cisplatin treatments (Fig. 3B). Moreover, when ERp44 was downregulated, Caspase3 and Bax which could promote apoptosis were increased, while Bcl-2 and Bcl-xl that could inhibit apoptosis were decreased. And the phenotype became more obviously with cisplatin treatment (Fig. 3C). Interestingly, we also observed cells present membrane swollen bulge form and bubbles blown from the membrane after the treatment of cisplatin, which was an important character of cell pyroptosis (Fig. 3D). Western blot showed the pyroptosis marker, active GSDME-N section, was increased when ERp44 was knocked down (Fig. 3E). Thus, ERp44 could reduce cisplatin sensitivity by influencing cell apoptosis and pyroptosis.

### Exosomes played an important role in chemosensitivity of NPC cells

**Fig. 4:**
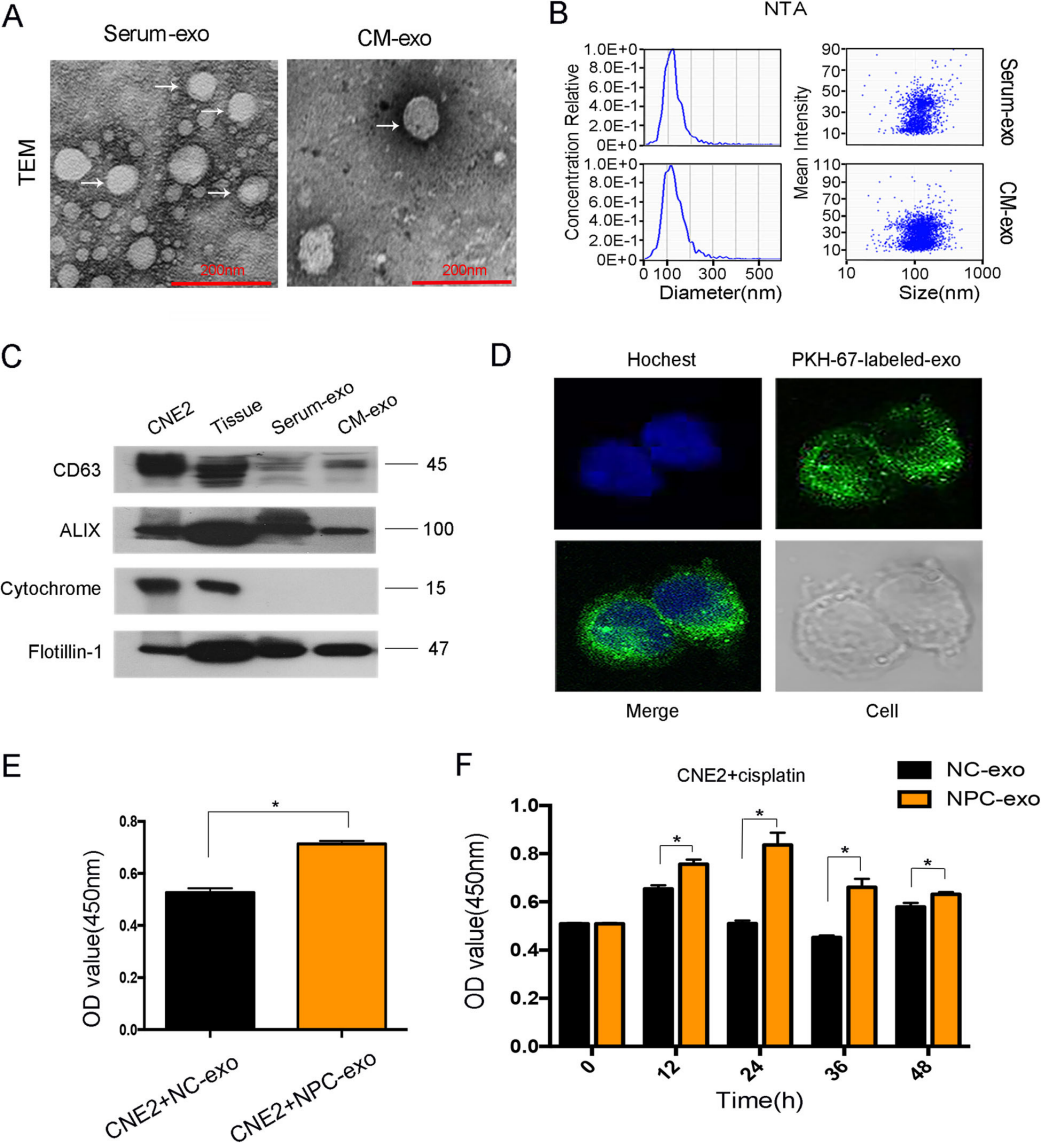
Exosomes played an important role in chemosensitivity of NPC cells. **A**: Representative images of exosomes from serum of NPC patients (serum-exo) or conditioned media of NPC cells (CM-exo) under Transmission electron microscopy (TEM). **B**: Nanoparticle tracking analysis was used to access the diameter of exosomes. **C**: Western blot was used to detect exosomal markers. Flotillin-1 was used as a loading control. **D**: Uptaken of exosomes by CNE2 cells was observed under confocal microscopy. Blue: Hoechst staining; green: PKH67-labeled exosomes. **E**: CCK8 was used to detect cell proliferation with the addition of NPC-derived exosomes. **F**: CCK8 was used to detect cell proliferation with cisplatin treatment after the addition of NPC-exosomes. *P < 0.05

Exosomes are discovered as a new system for cell-to-cell communication, but their role in NPC chemosensitivity remains unclear. We performed differential centrifugation to isolate exosomes from serum of NPC patients (serum-exo) or conditioned media of NPC cells (CM-exo). Under transmission electron microscopy, exosomes showed a structure of lipid bilayer membrane (Fig. 4A). Then we took nanoparticle tracking analysis and found the diameter of exosomes was around 100 nm(Fig. 4B). Western blot also confirmed our extraction. The markers for identifying exosomes, such as CD63 and ALIX were highly expressed in exosomes, while the negative control, cytochrome, was hard to find (Fig. 4C). More importantly, exosomes could be uptaken by NPC cells after the coculture (Fig. 4D). Then we investigated the function of exosomes on NPC proliferation. As shown in Fig. 4E, NPC-derived exosomes accelerated CNE2 proliferation. What’s more, with cisplatin treatment, cell viability was increased after the addition of NPC-exosomes compared with NC-exosomes (Fig. 4F). So NPC-derived exosomes could be secreted and uptaken by tumor cells to influence cell chemosensitivity.

### ERp44 was enriched in ERS-exosomes and could strengthen chemoresistance of NPC cells

**Fig. 5:**
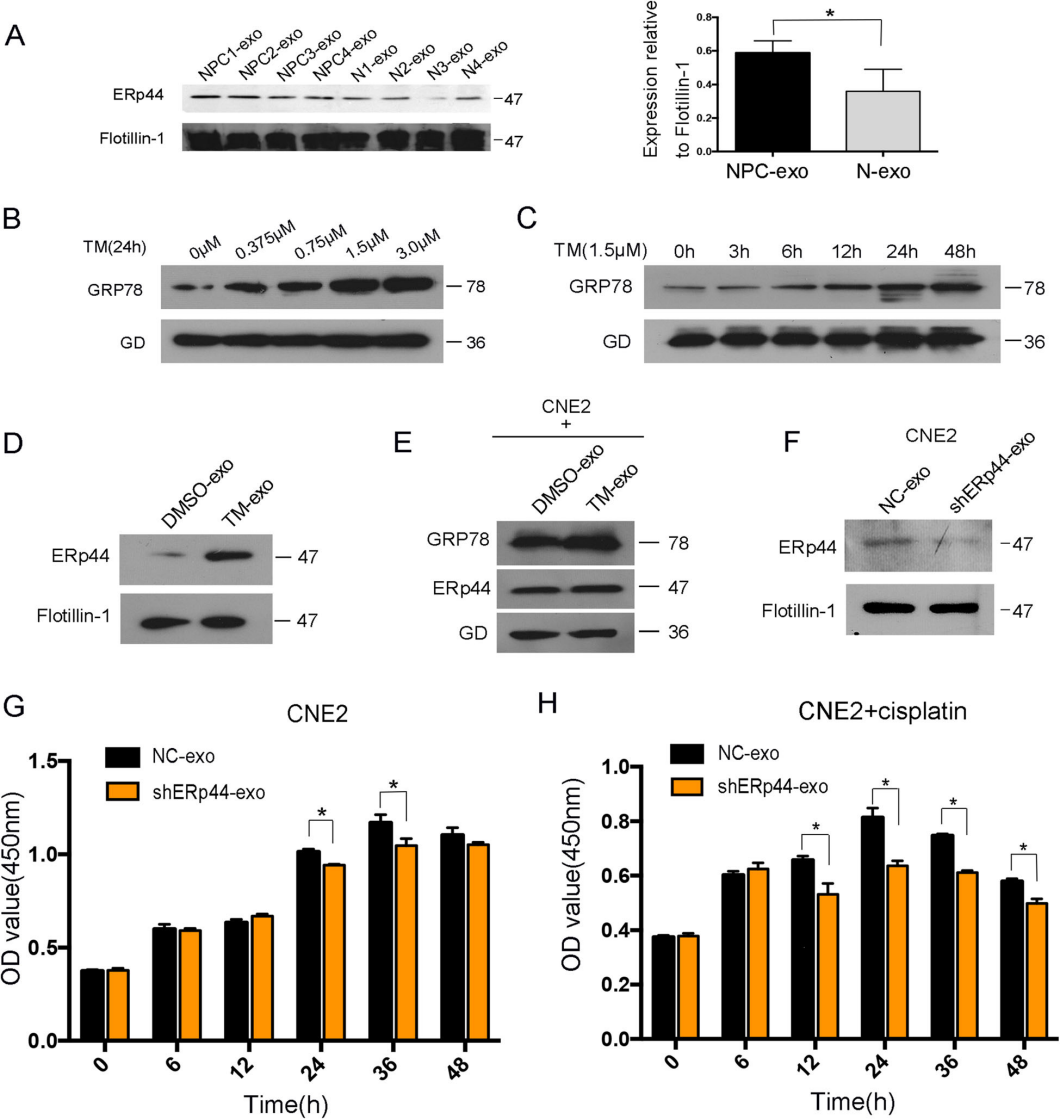
ERp44 was enriched in ERS-exosomes and could strengthen chemoresistance in NPC. **A**: Western blot was used to detect ERp44 expression in exosomes from NPC patients and normal volunteers. The histogram showed ERp44 expression relative to Flotillion-1. **B**: Western blot showed the expression of GRP78 with the treatment of different concentrations of tunicamycin (TM) for 24 h. **C**: Western blot showed the expression of GRP78 with the treatment of 1.5 μM TM for different time points (0 h,3 h,6 h,12 h,24 h,48 h). **D**: Western blot showed ERp44 expression level in exosomes with the treatment of TM (1.5 μM) for 24 h. **E**: Western blot showed the expression of GRP78 and ERp44 after the treatment of TM-exosomes, **F**: Western blot showed the expression of ERp44 in exosomes when ERp44 was knocked down. **G**: CCK8 assay showed that cell viability was decreased after the treatment of shERp44-exosomes. **H**: CCK8 assay showed chemosensitivity of CNE2 was increased with the treatment of shERp44-exosomes. * *P* < 0.05

Exosomes released by tumor cells can carry proteins, lipids, RNAs and DNAs to recipient cells to exert their function. Next, we wonder what ingredients in exosomes took effects. Exosomes were extracted from the serum of NPC patients and normal volunteers. Western blot showed compared with normal people-derived exosomes, ERp44 was highly expressed in NPC-derived exosomes (Fig. 5A). As ERp44 was overexpressed in ERS tissues, we hypothesized ER-stressed cells might also release ERp44-containing-exosomes and influence cell chemosensitivity. We used tunicamycin (TM) to induce ERS and found treating cells with 1.5 μM TM for 24 h generated the most effective expression of GRP78 (Fig. 5B-C). Western blot showed ERp44 was also increased in exosomes after 24 h TM treatment (Fig. 5D). So ERp44 was enriched in ER-stressed cells derived-exosomes. Cells were then co-cultured with exosomes and ERp44 was elevated in CNE2 once treated with TM-exosomes, accompanied with higher expression of GRP78 (Fig. 5E). The results illustrated that NPC cells could transmit ER stress signals through exosomes. To further rule out the role of ERp44, we knocked it down and collected exosomes (Fig. 5F). CCK8 assay showed cell viability was decreased after the treatment of shERp44-exosomes, and this treatment could increase chemosensitivity of CNE2(Fig. 5G-H). Taken together, under ERS, NPC cells produced ERp44-containing-exosomes, which could be transferred to adjacent cells and strengthen chemoresistance.

### ERp44 facilitated chemoresistance in vivo

**Fig. 6:**
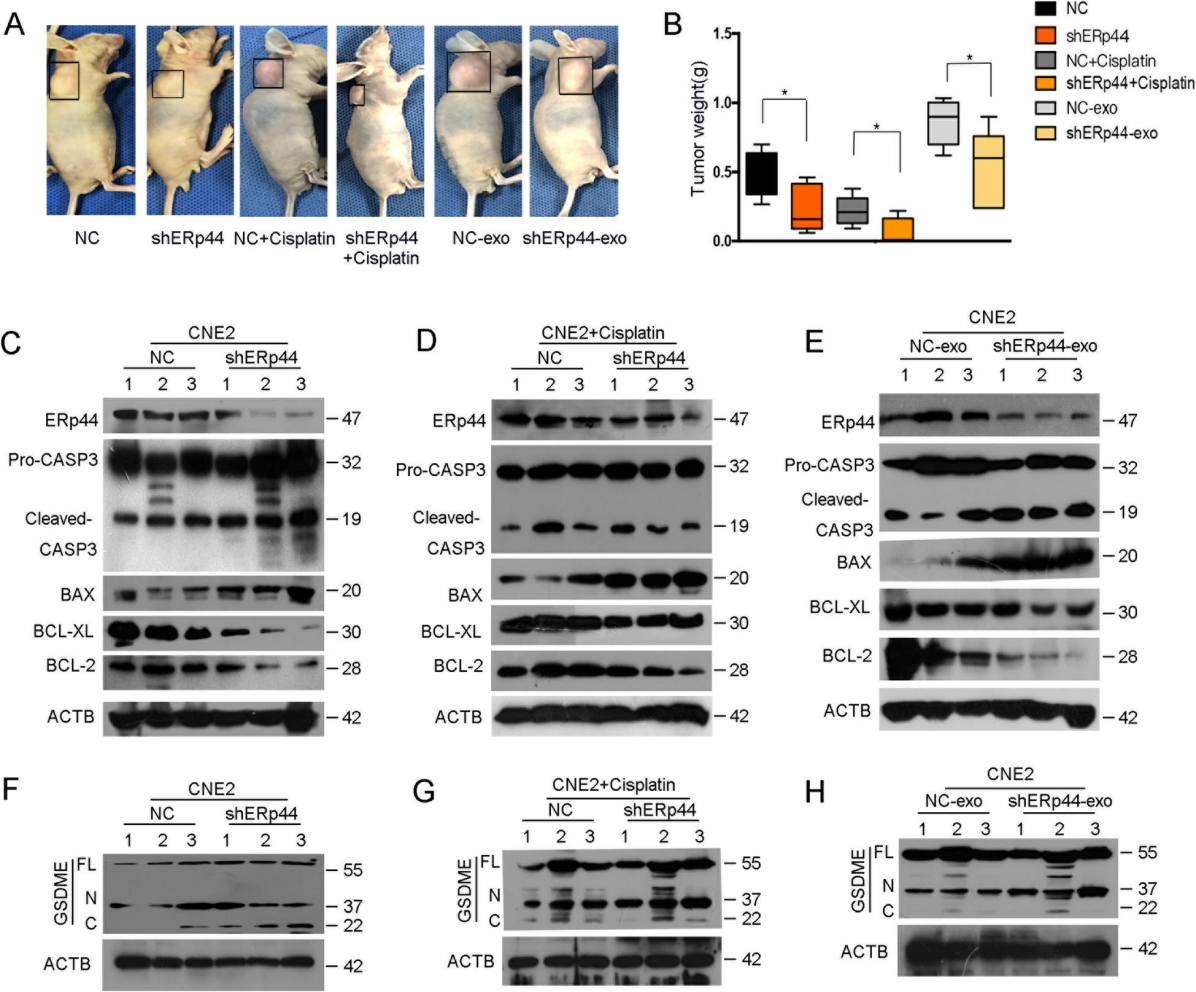
ERp44 facilitated chemoresistance in vivo. **A**. The first four groups represented that CNE2 cells transfected with shERp44 or NC were subcutaneously injected into the mice. After tumor formation, cisplatin was intraperitoneal injected every 2 days. The last two groups represented that CNE2 cells were subcutaneously injected into the mice, after tumor formation, shERp44-exosomes or NC-exosomes were intratumorally injected every 2 days. We showed the representative pictures of NPC xenografts in nude mice. **B**. The histogram showed the weight of tumors in different groups (*n* = 5 per group). **C**. Western blot showed the expression of apoptosis markers in tumors when ERp44 was knocked down. **D**. Western blot showed the expression of apoptosis markers in tumors after ERp44 was knocked down with cisplatin treatment. **E**. Western blot showed the expression of apoptosis markers in tumors with shERp44-exosomes treatment. **F**: Western blot showed GSDME expression in tumors after ERp44 was knocked down. **G**: Western blot showed GSDME expression in tumors after ERp44 was knocked down with cisplatin treatment. **H**: Western blot showed GSDME expression in tumors with shERp44-exosomes treatments. * *P* < 0.05

Finally, we determined whether ERp44 could facilitate chemoresistance in vivo. CNE2 cells transfected with shERp44 were subcutaneously injected into nude mice. After tumor formation, we intraperitoneal injected cisplatin every 2 days. As illustrated in Fig. 6A-B, tumor weight was smaller when ERp44 was knocked down. And ERp44-knocked down group exhibited significantly enhanced drug sensitivity. Moreover, when treated with shERp44-exosomes, tumors were smaller than control. So ERp44 in exosomes could strengthen chemoresistance of NPC cells. Western blot showed apoptosis markers were increased in shERp44 group with or without cisplatin treatment (Fig. 6C-D). What’s more, when treated with shERp44-exosomes, apoptosis markers expression were also increased (Fig. 6E). Finally, we found active GSDME-N section was increased in shERp44 group with or without cisplatin treatment, which illustrated the occurrence of pyroptosis (Fig. 6F-G). And it was also elevated after the addition of shERp44-exosomes (Fig. 6H). In a word, ERp44 could reduce cisplatin sensitivity by influencing cell apoptosis and pyroptosis in vivo.

## Discussion

According to National Comprehensive Cancer Network (NCCN) Guidelines, chemotherapy is a crucial part for NPC treatment [1]. Based on the previous studies, chemotherapy is beneficial to the overall survival of NPC patients and cisplatin is commonly a first choice [33]. However, resistance to chemotherapy is still an important factor for treatment failure [34, 35]. Here, we showed that exosomal ERp44 derived from ER stressed-NPC cells could strengthen cisplatin resistance. This might be a new regulatory mechanism of NPC chemosentivisity.

Studies reported that UPR has an essential influence on tumor progression [11]. It is activated in tissues as evident by the related markers, such as GRP78, PERK, ATF6 and IRE1α. GRP78, a master regulator in UPR, is reported to be overexpressed in tumors, including multiple myeloma and prostate cancer. It might acted as a modulator of cell adhesion marker [36, 37]. In HNSC, GRP78 was significantly elevated and could enhance tumor malignancy [38]. In accordance with these research, we confirmed that GRP78 was highly expressed in NPC, which might illustrate ERS is activated. Moreover, we also found ERp44 was elevated in ER stressed tissues (Fig. 1A-C). ERp44 was reported to be abnormally expressed in tumors and we have confirmed that it participated in promoting malignant phenotype of NPC [22]. But its role on NPC chemosentivisity remains unclear.

The relationship between ERS and drug resistance caught our attention. Thakur at al. reported that inhibition of ERS-mediated autophagy enhanced the effectiveness of chemotherapeutics on pancreatic cancer [39]. In ovarian cancer, an increased level of GRP78 contributed to cisplatin resistance [40]. One of the most important findings of our research was that ERS was up-regulated in NPC and positively correlated with ERp44. More importantly, when ERp44 was knocked down, cells became more sensitive to cisplatin (Fig. 2). So we took further studies to investigate the detailed mechanism between ERp44 with cisplatin resistance.

Apoptosis, one of the pathways of programmed cell death, is an emerging target for better treatment [41]. In B-cell malignancies, overexpression of antiapoptotic proteins was associated with treatment resistance [42]. ERp44 could also influence cell apoptosis. In Hela cells, knockdown of ERp44 caused remarkable cell apoptosis [43]. In oral squamous cancer cell carcinoma (OSCC), when ERp44 was downregulated, cell proliferation was reduced, while apoptosis was significantly induced [19]. NF-κB has been described to be involved in cell apoptosis and platinum-based chemotherapy resistance [32]. In nonsmall-cell lung cancer (NSCLC), NF-κB was a potential therapeutic target in cisplatin-resistant cells [44]. In our research, we found when ERp44 was downregulated, cells became more sensitive to cisplatin and could inhibit NF-κB to promote cell apoptosis (Fig. 3A-C). Besides apoptosis, many other cell death forms including pyroptosis have been identified these years [45]. It has characteristics of cell swelling and rapid plasma membrane lysis. GSDME, identified as DFNA5 (Deafness, Autosomal Dominant 5), could be cleaved specifically by caspase-3 and generates a GSDME-N fragment to mediate pyroptosis [46]. We also found active GSDME-N section was increased after ERp44 was knocked down (Fig. 3D-E). Thus, ERp44 could reduce cisplatin sensitivity by influencing cell apoptosis and pyroptosis.

Exosomes are a subset of phospholipid-enclosed vesicles released by cells and are present in body fluids. They are produced by tumor cells and carry biological materials to influence tumor progression [47]. Our previous studies confirmed exosomes play important roles in NPC [26]. In this research, we also collected exosomes successfully and validated that NPC-exosomes could promote cell proliferation. Moreover, it increased cisplatin resistance of NPC cells (Fig. 4). ERS could also promote tumor cells to release exosomes. Xiaoli Yao et al. reported that under ERS, breast cancer cells produced exosomes that could up-regulate PD-L1 in macrophages and promote immune evasion [25]. According to our data, ERS was activated in NPC. We hypothesized exosomes might transmit UPR-associated signals to cells around. Subsequently, we treated cells with TM to mimic ERS and collected ER-stressed cells derived-exosomes (Fig. 5 B-C). The contents of exosomes are complex and could be transferred to receipt cells. Under ERS, liver cancer cells could secrete miRNA-23a-3p-containing-exosomes to macrophages and make tumor cells escape from antitumor immunity [28]. In our research, we found ERp44 was highly expressed in NPC derived exosomes. After TM treatment, its expression level became higher (Fig. 5A, D). What’s more, NPC cells could transmit ER stress signals through exosomes (Fig. 5E). Then, we also showed exosomes that had low ERp44 could inhibit CNE2 proliferation and increase cisplatin sensitivity (Fig. 5G-H). Our results were further confirmed in vivo (Fig. 6). So under ERS, tumor cells produced ERp44-containing-exosomes, which could be transferred to adjacent cells and strengthen chemoresistance. Our findings provided a new insight of ERS-exosomes in tumor chemoresistance.

## Conclusion

Taken together, we found ERp44 was elevated in ER-stressed tissues and could reduce cisplatin sensitivity by influencing cell apoptosis and pyroptosis. Moreover, under ERS, NPC cells secreted ERp44-containing-exosomes to strengthen cell chemoresistance. These results indicated that ERp44 takes an inevitable role in NPC chemoresistance and might act as a novel treatment target.

## Data Availability

All data generated or analyzed during this study are included in this published article.

## Abbreviations

NPC: nasopharyngeal carcinoma
ER: Endoplasmic reticulum
ERS: Endoplasmic reticulum stress
UPR: Unfolded Protein Response
GRP78: Regulated Protein 78
ERp44: ER resident protein 44
PDI: Protein disulfide isomerase
IHC: Immunohistochemistry
TEM: Transmission electron microscopy
NTA: Nanoparticle tracking analysis
HNSC: Head and Neck squamous cell carcinoma
TCGA: The Cancer Genome Atlas
NF-κB: Transcription factor nuclear factor kappa-B
TM: Tunicamycin
OSCC: Oral squamous cancer cell carcinoma
